# Use and Acceptance of Smart Elderly Care Apps Among Chinese Medical Staff and Older Individuals: Web-Based Hybrid Survey Study

**DOI:** 10.2196/41919

**Published:** 2023-06-13

**Authors:** Jieting Zhu, Huiting Weng, Peng Ou, Lezhi Li

**Affiliations:** 1 Department of Vascular Surgery Second Xiangya Hospital Central South University Changsha China; 2 Clinical Nursing Teaching and Research Section Second Xiangya Hospital Central South University Changsha China; 3 Department of Anesthesiology Second Xiangya Hospital Central South University Changsha China; 4 Xiangya Nursing School Central South University Changsha China

**Keywords:** smart elderly care app, mobile health, smartphone

## Abstract

**Background:**

With the advent of China’s aging population and the popularization of smartphones, there is a huge demand for smart elderly care apps. Along with older adults and their dependents, medical staff also need to use a health management platform to manage the health of patients. However, the development of health apps and the large and growing app market pose a problem of declining quality; in fact, important differences can be observed between apps, and patients currently do not have adequate information and formal evidence to discriminate among them.

**Objective:**

The aim of this study was to investigate the cognition and usage status of smart elderly care apps among older individuals and medical staff in China.

**Methods:**

From March 1, 2022, to March 30, 2022, we used the web survey tool Sojump to conduct snowball sampling through WeChat. The survey links were initially sent to communities in 23 representative major cities in China. We asked the medical staff of community clinics to post the survey link on their WeChat Moments. From April 1 to May 10, 2022, we contacted those who selected “Have used a smart elderly care app” in the questionnaire through WeChat for a request to participate in semistructured interviews. Participants provided informed consent in advance and interviews were scheduled. After the interviews, the audio recordings were transcribed into text and the emerging themes were analyzed and summarized.

**Results:**

A total of 810 individuals participated in this study, 54.8% (n=444) of whom were medical staff, 33.1% (n=268) were older people, and the remaining participants were certified nursing assistants (CNAs) and community workers. Overall, 60.5% (490/810) of the participants had used a smart elderly care app on their smartphone. Among the 444 medical staff who participated in the study, the vast majority (n=313, 70.5%) had never used a smart elderly care app, although 34.7% of them recommended elderly care–related apps to patients. Among the 542 medical staff, CNAs, and community workers that completed the questionnaire, only 68 (12.6%) had used a smart elderly care app. We further interviewed 23 people about their feelings and opinions about smart elderly care apps. Three themes emerged with eight subthemes, including functional design, operation interface, and data security.

**Conclusions:**

In this survey, there was a huge difference in the usage rate and demand for smart elderly care apps by the participants. Respondents are mainly concerned with app function settings, interface simplicity, and data security.

## Introduction

### Background

Owing to the advanced computing technology and greater connectivity using cellular network architecture, smartphones are currently one of the main forms of media for the democratization and dissemination of mobile health [1]. According to a 2021 report on mobile phone use in China [2], 70.29% of older adults spend more than 2 hours online each day and 31.80% spend more than 4 hours online daily. Older adults show subjectivity and active learning in the ability to use mobile phones, and they will take the initiative to acquire health-related knowledge, cook, and dance on the internet [3]. The development of smartphones has been accompanied by an increase in the number and usage of mobile apps. These software programs running on devices such as smartphones can be preinstalled or downloaded from app markets (eg, Google Play Store, Apple App Store, Android Phone Store). Some of these apps focus primarily on health and health care.

Over the past few decades, smartphones have fundamentally changed our daily lives. In terms of health, older adults now have more opportunities to obtain health knowledge and improve the convenience of their medical treatment using apps. Several studies have demonstrated the effectiveness of mobile health interventions in the management of lifestyle factors (eg, improving dietary habits, quitting smoking, and increasing physical activity) and chronic diseases (eg, diabetes, Parkinson disease, high blood pressure). This evolution has affected the relationship between general practitioners and patients [4-6]. Recent studies have shown that patients view mobile health apps as useful complementary tools for self-monitoring and self-management of their health, albeit with some limitations about charge problems and the legality of electronic prescribing [7,8]. A qualitative study involving French general practitioners highlighted contradictory narratives surrounding mobile health app prescriptions or patient use of apps [9]. In France, health apps must be considered “medical devices” and must be evaluated by the National Committee for the Evaluation of Medical Devices and Health Technologies (Annex) to be reimbursed [8].

### Current Status of Smart Elderly Care Apps in China

In China, reports of the inconvenience experienced by older adults due to their inability to use smartphones attracted the attention of society as a whole in 2020 [10]. Therefore, in 2021, the Ministry of Industry and Information Technology of the government launched a one-year “Special Action for Aging and Barrier-Free Transformation of Internet Applications” [11]. This report found a low level of use of internet applications by the older population due to complex interfaces and difficult-to-use operations; thus, older adults typically avoid using these applications and do not know how to use them. There are common problems such as a lack of text description in pictures, difficulty in operating verification codes, and incompatibility between related functions and devices. At the same time, the report also clarified the standards for suitable features of internet applications for older individuals, including more products with features such as large fonts, large icons, and high-contrast text. Enterprises are encouraged to introduce models with simple interfaces and convenient operations, and to realize various barrier-free functions such as one-key operations and text input prompts. In China, improving dialect recognition facilitates the use of smart devices by older individuals who do not speak Mandarin. In addition, in view of the problem that there are many mandatory advertisements in current internet applications, which can easily mislead older individuals, internet websites and mobile apps will need to include versions that are friendly to the older population and accessible, such as no longer including advertising plug-ins, especially for payments. There will be no inductive keys for such operations, enabling various special groups to use these applications conveniently and safely.

In 2020, China’s older population (aged>65 years) was approximately 177 million, accounting for 12.64% of the national population, which exceeded the international standard of 7% for aging countries; the population of people over 60 years old was approximately 268 million, accounting for 18.87% of the national population [12]. In addition, the increase in the consumption power of the older population has promoted the growth of the demand for elderly care services. The older age care model implemented in China is mainly the “9073” model; that is, 90% of older adults are cared for by their families, 7% enjoy community home-based care services, and 3% enjoy institutional care services. Therefore, from 2020 to 2050, there has been a huge demand for home medical and nursing care among China’s older population [13].

Existing smart elderly care apps mainly focus on six areas: chronic disease management, home health management, personalized health management, internet health consultation, door-to-door life care, and information-based health records [14]. In 2010, the Office of Aging of the Chinese Government proposed the informatization of elderly care services [15]. To date, there are three main modes [16]. The first includes public welfare elderly care apps developed and operated by provincial/municipal governments, such as Beijing Tong, Hefei Elderly Care Certification, and Langfang Medical Care Serve. The main services provided by this type of app include endowment insurance, information on nearby hospitals, appointment registration, and door-to-door medical care services. The second mode is supported by the provincial/municipal government, including the public welfare pension app developed and operated by enterprises, such as Shunde Smart Pension and People’s Livelihood Smart Pension. In addition to the above-mentioned functions, such apps also provide charging services such as nearby nursing homes, online drug stores, and paid health consultations. The third category is for-profit elderly care apps independently developed by enterprises, such as Huiying Consumer Pension and Ankang Human Society. Most of the services provided by such apps are paid services and there are security risks.

The objective of this study was to investigate the cognition and usage status of smart elderly care apps among older individuals and medical staff in China. Secondary goals were to collect the types of health apps expected by older adults and analyze the factors associated with the use of these apps based on the sociodemographic, geographic, and medical characteristics of the study population.

## Methods

### Questionnaire Design

For the purpose of the study, a specific questionnaire was developed and distributed to the research participants. We searched for smart elderly care apps from Google Play Store, Apple App Store, and Android App Store, and designed a questionnaire based on the functions provided in the existing apps and the problems encountered by older adults when using them. The questionnaire consisted of 36 questions and was estimated to take approximately 5 minutes to complete. The questionnaire was presented to specialists (5 health care workers, 5 community workers) and then tested on 10 older adults (aged>60 years). Considering their suggestions and recommendations, the questionnaire was revised to make it as understandable and relevant as possible. Before the survey, we introduced the participants to the background of the survey. The questionnaire was completed voluntarily without any compensation. If respondents did not agree with the options listed, they could choose another option and write their answers in the “Remarks” column. The questionnaire covered demographic information as well as the usage status, perceptions, and needs related to smart elderly care apps.

### Survey Platform and Methodology

WeChat has become one of the largest mobile traffic platforms in China, which offers many services, including messaging, toll-free calls, browsing and publishing of instantly shared information, and mobile payments. Research shows that approximately 79.87% of older adults in China use WeChat as their main social tool [12]. The network also enables researchers to manage questionnaires through WeChat.

From March 1, 2022, to March 30, 2022, we used the web survey tool Sojump to conduct snowball sampling through WeChat. The survey links were initially sent to community health service clinics in 23 representative major cities in China. We asked the staff of these communities to post the survey link on their WeChat Moments.

From April 1 to May 10, 2022, we contacted those who selected “Have used a smart elderly care app” in the questionnaire through WeChat for consent to schedule brief semistructured interviews. The content of the interviews is shown in Textbox 1. Video calls were used to conduct the interviews and were screen-recorded. Participants were interviewed for approximately 10-15 minutes, depending on the content of the interview outline. After the interviews, the audio recordings were transcribed into text and summarized into themes.

**Semistructured interview outline.**

1. Would you like to use a smart elderly care app?

2. What concerns are affecting your use of a smart elderly care app?

3. What concerns are affecting your LACK OF willingness to use a smart elderly care app?

4. What function do you think the app should include?

5. Should there be a fee for the app? Why or why not?

6. Which app functions would you accept to pay for? How much?

7. If there is a service to find a doctor/nurse/caregiver in the app, will you use it? Why or why not?

### Ethics Approval

The study was approved by the Ethics Committee of the Second Xiangya Hospital, Central South University (ID 2022-S570).

### Data Analysis

The questionnaire data were analyzed by SPSS version 23.0. Categorical variables are expressed as counts and percentages. The *χ^2^* test was used to assess differences between groups. The generalized logic model was used to obtain the odds ratio (OR) and its 95% CI. We conducted a univariate analysis to analyze the OR of the potential association between demographic factors and willingness to use a smart elderly care app. We then input all significant factors into a multivariate model to obtain the multivariate-adjusted OR. Questionnaires with missing values were excluded from the multivariate analysis. Statistical significance was indicated with *P*<.05.

The audio recordings from the interviews were transcribed verbatim within 24 hours immediately after the interview and then combined with field notes to collate all verbal and nonverbal information from the interviewees into textual material. With the assistance of the qualitative research software NVivo 11.0 (QSR International), two researchers jointly collected, organized, stored, classified, summarized, and named the data; coded all the documents; and finally separated out the themes and developed them into subthemes. The data were analyzed using the Colaizzi 7-step analysis method [16,17]: (1) carefully read all transcripts, (2) extract significant statements, (3) encode recurring and meaningful ideas, (4) summarize coded ideas, (5) write a detailed description without omission, (6) identify similar viewpoints and sublimate the subject concept, and (7) return to the research object for verification. In the process of data analysis, if two researchers had different opinions, members of the research group discussed and reviewed them. Data were “saturated” when no new themes emerged from the data analysis.

## Results

### Results of Questionnaire

**Figure 1:**
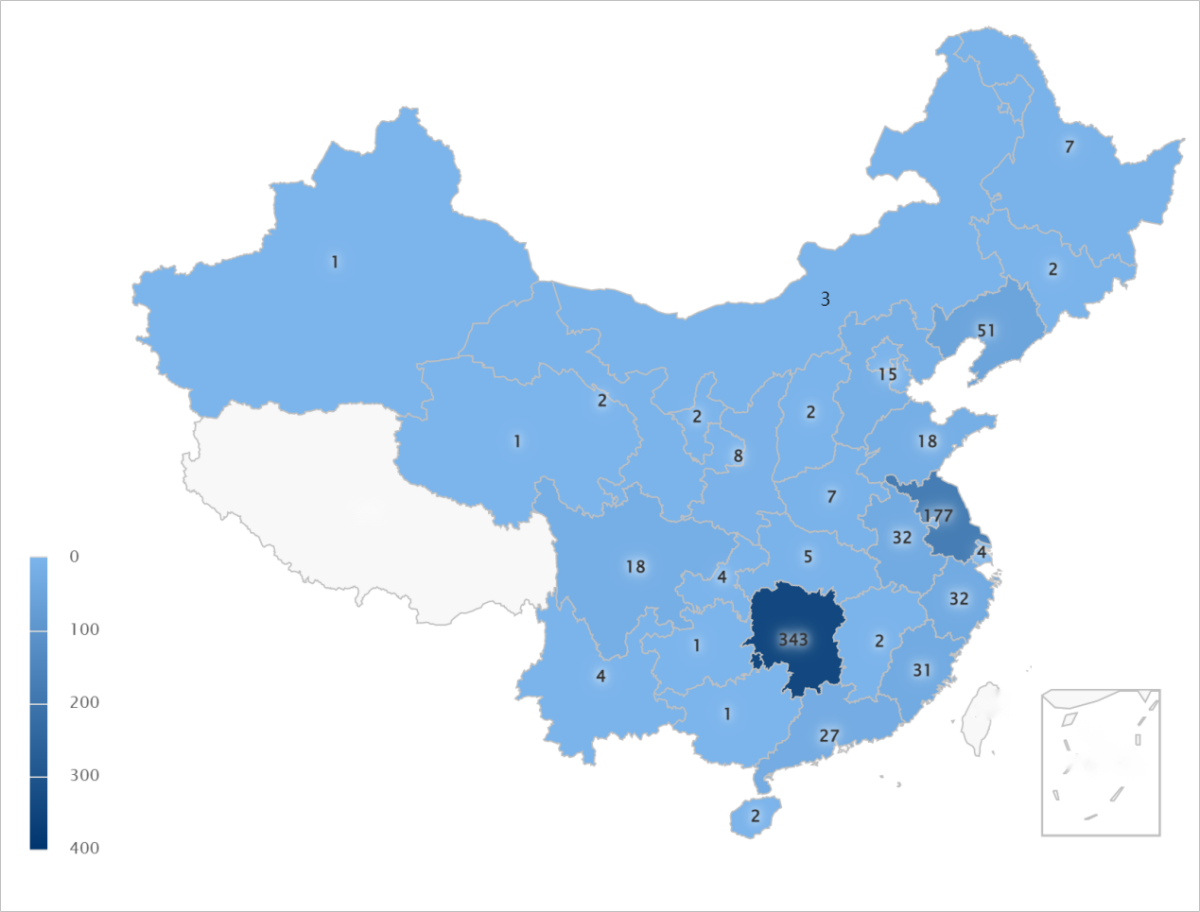
Distribution of the respondent sample in China by province.

A total of 810 individuals participated in this study, and their provinces of residence are shown in Figure 1. Demographic characteristics and main questionnaire responses are summarized in Table 1.

**Table 1:** Demographic characteristics of the survey sample (N=810).

Characteristic			Respondents, n (%)
**Gender**			
	Male	244 (30.1)	
	Female	566 (69.9)	
**Age (years)**			
	<30	144 (17.8)	
	30-60	380 (46.9)	
	≥60	268 (33.1)	
**Socioprofessional category**			
	Medical stuff	444 (54.8)	
	Certified nursing assistant	23 (2.8)	
	Community worker	75 (9.3)	
	Retired	268 (33.1)	
**Have you used a smart elderly care app?**			
	Yes	490 (60.5)	
	No	320 (39.5)	
**Have you recommended patients to use such an app?** **(for medical staff only, n=444)**			
	Yes	219 (49.3)	
	No	225 (50.7)	
**Is it legal to prescribe medication for older adults through an app?**			
	Yes	433 (53.5)	
	No	87 (10.7)	
	I don’t know	290 (35.8)	
**Is it legal to guide older adults on diet/exercise through an app?**			
	Yes	549 (67.8)	
	No	59 (7.3)	
	I don’t know	202 (24.9)	
**I think the future of smart elderly care apps is?**			
	Good	615 (75.9)	
	Just so-so	155 (19.1)	
	Not good	40 (4.9)	
**Will you recommend older adults to use such apps in the future? (for medical staff only, n=444)**			
	Yes	113 (14.0)	
	Probably	223 (27.5)	
	No	54 (6.7)	

Among the respondents, 54.8% (444/810) were medical staff, 33.1% (268/810) were older adults (≥60 years), and the rest were certified nursing assistant (CNAs) and community workers. Among the 268 older adults, 135 (50.4%) were receiving in-home care, 67 (25.4%) were living in nonprofit nursing homes, and 66 (24.6%) were living in commercial nursing homes, which is in line with existing survey reports in China [15]. The types and characteristics of smart elderly care apps used by all participants are summarized in Table 2.

**Table 2:** Types and characteristics of smart elderly care apps used by participants (N=810).

App	Participants, n (%)	Function and features
None used	562 (69.8)	Not applicable
Yuyao Smart Elder Care	102 (12.6)	Used in conjunction with smart wearable devices, real-time monitoring, emergency calls, data analysis, and early warning
Honghua Healthcare	92 (11.4)	Provides communication, entertainment, home delivery of meals, assistance in bathing, housekeeping
Health Butler	83 (10.3)	Online disease consultation and appointment registration, door-to-door life care, intermediary services, housekeeping services
Wenzhou Elder Care Service Platform	78 (9.6)	Apply for endowment insurance online, receive pensions, learn about the latest national policies, and provide university education for the elderly
Wellness Care	74 (9.1)	Health care, shopping, emergency assistance, home care
Beijing Tong (Personal Edition)	56 (6.9)	Provide family doctor signature, health assessment, door-to-door medical care, life care, and nursing skills training for community residents
Others	43 (5.3)	Various

Among the 810 participants, 60.5% (n=490) had used a smart elderly care app on their smartphone. Among the 444 medical staff who participated in the study, the vast majority (n=313, 70.5%) had never used a smart elderly care app, although 34.7% of them recommended such apps to patients and half of them (n=219, 49.3%) recommended disease-related apps to patients, such as hypertension and diabetes-related apps.

**Figure 2:**
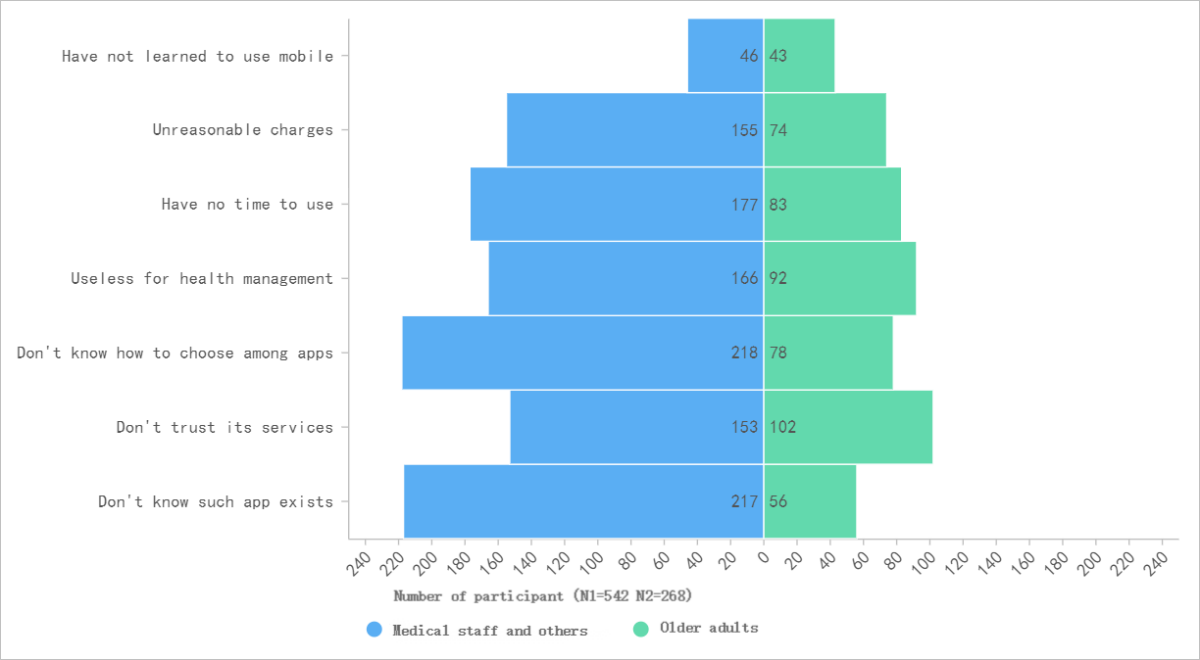
The factors that influence medical staff and older adults to use smart elderly care apps.

We further evaluated the factors that influence medical staff and older adults to use smart elderly care apps. The reason why most participants had never used the apps is that they did not know they existed. Of those who did use the apps, they found the quality of the app to be mixed, and they did not know how to choose an app for their purposes. The detailed reasons for app use are provided in Figure 2.

Notably, the vast majority of participants (770/810, 95.1%) believe that the future of smart elderly care apps is very bright. Only 40 (4.9%) believe that they will never use any app related to elderly care in the future. The main reasons given for lack of use were related to concerns about disinformation and data security. This is a strong signal to researchers, software developers, and businesses that we should take steps to develop apps that meet the needs of both older adults and health care workers.

### Main Themes

**Figure 3:**
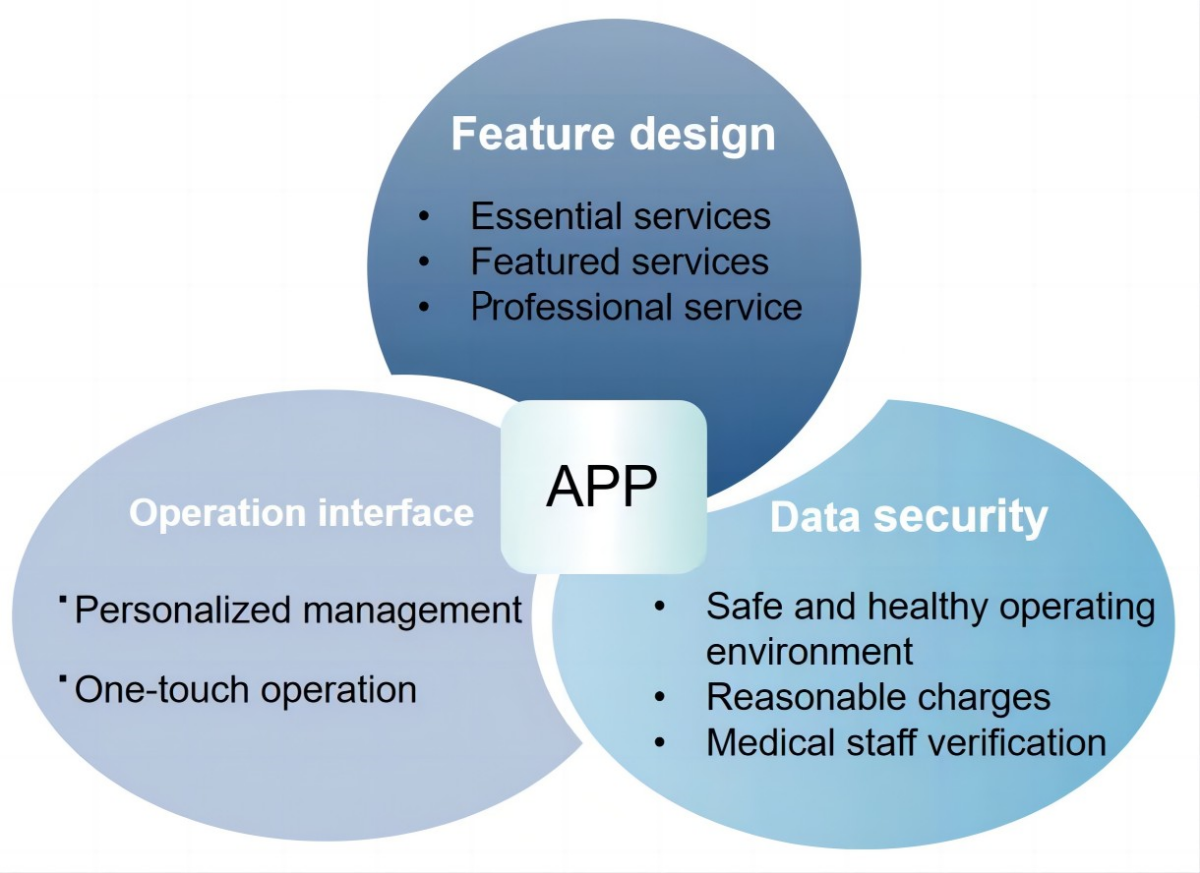
Themes that emerged from the interview content.

A total of 542 medical staff, CNAs, and community workers completed the questionnaire, among whom only 68 (12.6%) had reported using a smart care app for older individuals. We interviewed 23 individuals of the sample until reaching “saturation” of information, which means that no new theme emerged from the responses. Based on analysis with NIVIVO software, three themes and eight subthemes emerged from the qualitative analysis, as presented in Figure 3.

### Theme 1: Functional Design That Meets the Needs of Older Adults

#### Essential Services (Living Care and Health Care)

Almost all of the respondents mentioned that they hope that the main functions of the app include life care recommendations for older individuals, such as diet, cleaning, shopping, housework, and other services, as well as special care services such as escort review and escort services. Through the app, they can learn about health care, pension policies, government news, and other information.

If my food, clothing, housing, and transportation can be facilitated on the app, I would be happy to use it.

I hope to see content about health and wellness, health science, etc on the app

Self-care ability of older individuals declines, and life care should give priority to caring for the diet, body waste, sleep, bodily cleaning, etc, which can be through teaching skills or the provision of services.

If I need it, I hope I can contact the escort or short-term nanny through the app to provide me with housekeeping services.

#### Featured Services (Chronic Disease Guidance or Door-to-Door Medical Care)

Older individuals with some diseases or reduced mobility may require an escort to accompany them outside of the home. It is also necessary to provide disease prevention, rehabilitation care, mental health services, and hospice care for older individuals and to establish health records.

Hypertension, hyperglycemia, and hyperlipidemia are common among elderly individuals. I hope that there will be online questions about medical care or appointments for medical care.

I need to take a variety of medicines, some of which are prescription drugs. It is very inconvenient to go to the hospital every time to prescribe the medicine. I hope that the medicine can be delivered to my home and a pharmacist will guide the medicine online.

The sequelae of cerebral infarction made my activities very inconvenient. I need to do rehabilitation treatment regularly. If I can make an appointment with a therapist for on-site services, it can save me a lot of trouble and help me a lot.

#### Professional Services for Older Adults With Special Needs

The care of older individuals with dementia is a difficult issue. An elderly care app can recommend suitable medical and nursing institutions and can provide extended services for some older people with other needs, such as providing legal advice; assistance and maintenance; information about property, marriage, and other legal rights; and other consulting services. They can also provide older adults with information about colleges, lectures, learning and training programs, reading, and other available services for the older population. Services such as mutual assistance between neighbors, heart-to-heart exchanges, and spiritual comfort are also important.

I hope to be able to make reservations and recommend elderly day service centers and activity venues through the app, so that we can have fun and make friends in our old age.

A section should be designed, through which you can handle subsidies for elderly care services, bed reservations in nursing homes, retrofits for aging, and community activities for elderly individuals.

### Theme 2: User-Friendly Operation Interface for Older Adults

#### Personalized Management

The needs of each older person are different. With age, there will inevitably be changes such as memory loss, blurred vision, and unresponsiveness. Adjusting the operation interface and designing functions of the app according to the actual situation of the individual user would make it more convenient for older adults to use the app.

Designing navigation features, especially to navigate to the hospital and back home.

Design a model for the older individual; the words should be large, clear, and look effortless.

It can be broadcast by voice or recognized by voice, and it is best to be able to distinguish dialects.

Be sure to have a voice notepad so I can remember what I’m going to do.

#### One-Touch Operation or Shortcut Keys

Older individuals usually have poor eyesight and inflexible hands. Therefore, designing a simple and easy-to-operate interface with few functions is the unanimous demand of older individuals, especially making sure the medical section is concise and clear.

The elderly are most afraid of accidents. We must design a simple “foolproof” operation that enables the elderly to quickly call for help and seek help.

Although the elderly have begun to use smartphones during the epidemic, the functions are too complicated for the elderly to operate, and the interface must be simple.

The simpler it is, the better it will be used. If it is complicated, you will not be able to learn it, and you will not want to learn it.

### Theme 3: Data Security

#### Safe and Healthy Operating Environment

Data security and privacy protection were the main concerns of the respondents. They hope that smart elderly care apps will be operated and supervised by the government or nonprofit institutions such as communities and public hospitals.

The reason why I dare not use various software is because I am afraid of being cheated out of money, such as telecom fraud or some unwilling payment, etc.

I do not trust most apps on the market. Even if the functions are complete, unless it is an app run by the government or the community, I should not use it.

I am very worried about whether my personal information will be leaked if I use the app. Many health issues involve privacy.

#### Reasonable Charges

Respondents did not advocate for free services; they believe that paid services are often better. They hope to combine various methods to provide a certain degree of unpaid or volunteer services to attract social volunteers to join and participate. However, it is also necessary to retain paid services, make them profitable, and use them for later maintenance and improvement of the app.

There can be a free part, and users need to be told what is the free part so that users can use it more at ease and can selectively use paid items.

There are clear charging standards, such as door-to-door medical services, the price can be set according to the degree of difficulty, and try not to have secondary charges.

Each older person’s economic situation is different. My retirement salary is very high. I prefer high-end and high-quality services, and I can choose by myself.

It can be divided into free services, compensation services, and paid services. For some people who need special care, the government will purchase services and provide a certain percentage of services that benefit the people.

#### Medical Staff Verification

In the life service and medical service section of the app, the work qualifications, areas of expertise, charging standards, and service content of service providers need to be standardized. The identities of medical staff, caregivers, and volunteers need to be identified and confirmed. Nonmedical staff cannot provide diagnosis and treatment services, and medical staff cannot provide medical services outside the scope of practice. Apps need to provide funding certification and audit services.

I have diabetes. If I use the app for diabetes consultation, I hope a professional endocrinologist will answer my questions.

I need professionally trained caregivers to answer my questions or come to my house.

Volunteers should be trained before they take up their jobs, and they need app real-name authentication. If they are bad people, then our safety will not be guaranteed.

## Discussion

### Principal Findings

In this study, 39.5% (320/810) of the sample surveyed had used a smart elderly care app, 50.7% (411/810) were willing to use it in the future, and only 9.8% (79/810) were reluctant to use such apps. This shows that in China, there is a great demand among older adults and elderly care–related personnel (eg, medical staff, community workers) for smart elderly care apps. Due to China’s previous one-child policy, more and more middle-aged people need to support two or more older people (parents and grandparents) alone. However, under the pressure of work and caring for children, they have no spare energy to take care of older individuals. Therefore, smart elderly care apps can save time and energy for younger generations, which is very beneficial.

Intelligent care for older adults was first proposed by the British Life Trust, formerly known as the “Intelligent Older System” [18], which refers to overcoming the shortcomings of the traditional care model that is constrained by time and space. The emergence and spread of the Internet of Things, reaching communities and medical institutions, form an organic whole to improve the quality of elderly care services. The literature shows that most provinces in China have begun to develop elderly care apps, but these pilots are generally concentrated in cities rather than rural areas, and well-developed pilots are mainly concentrated in developed areas such as Beijing, Shandong, and Shanghai. Intelligent elderly care in the central and western regions started late and has a low level of development [19,20]. Our results also show that in economically developed regions (such as Shanghai and Zhejiang), more people use smart elderly care apps and more people from these regions filled out the questionnaires (Figure 1).

Our research shows that a complete smart elderly care service app can fully integrate resources for older adults, including the integration and development of the older population ecological chain, building a presale service promotion system, and creating an in-sale service tracking system and an after-sale service evaluation system. Its functions should include medical treatment, health management, nourishment, eating, shopping, travel, and entertainment. Thereby, a new model of elderly care will be formed in which the government, operators, older individuals, children, service providers, housekeepers, and others will participate together. Such apps will be suitable for home care, community care, institutional care, and other application scenarios. This is consistent with the research of He and Zhan [21] and of An [22], who argued that smart elderly care apps should be suitable for various scenarios and roles, with joint participation of caregivers, older individuals, medical staff, intermediaries, and nursing home providers.

This study shows that older adults in China are very concerned about the ease of use of apps. Many older individuals have blurred vision and cannot use electronic products for a long time. An app interface with many function keys, complicated operations, and many pop-up windows would not be conducive to use by older individuals. In addition, older individuals are more prone to accidents such as falls and sudden illnesses. Therefore, they hope that a smart elderly care app can provide a one-key emergency calling function to immediately call their preset contacts. Paradis et al [7] also suggested that compared with young people, older people use fewer health management apps and pay more attention to their own safety. They also want to get effective exercise while keeping themselves safe.

In addition, the greatest concern of participants when using the app was privacy. When the functions of the app involve positioning, online payment, disease information, and personal information, the participants expressed great concern, and they were afraid of data leakage and the potential consequences. Some app functions will use this information, especially online consultations and on-site medical services, which require medical staff to conduct a qualification review when registering on the app, and they need to upload their doctor/nurse license to prove their identity. Older people are more worried about the safety of online payments. In China, people typically use Alipay or WeChat for online payments [23]. They need to perform real-name authentication on Alipay or WeChat and link a bank card in their own name to make payments. This cumbersome process requires the assistance of their dependents or family members to complete successfully. Additionally, when using online payments, older adults worry about being scammed or overpaying. These factors will affect their choice of app. However, this point is rarely considered in the existing smart elderly care apps that are available in China. Although information leakage caused by apps is very common, older adults lack awareness of information leakage and telecom fraud. Therefore, the occurrence of such problems should be considered when designing the functions of the app, especially the payment functions.

This leads to another question worth considering: should the development and operation of smart elderly care apps be carried out under the guidance or support of the government? In China, smart elderly care has begun to be valued and vigorously supported by the government, and an increasing number of products have been developed. The participation of the government often means more credibility. Therefore, the development and operation of many apps are closely related to the government. For example, Beijing Tongtong and Wenzhou Elder Care Service Platforms are apps developed by enterprises with the support of the government. However, due to the participation of the government, the functions of the apps are limited. Unofficial services such as online shopping, online food delivery, and intermediaries are often unavailable. Wan et al [24] mentioned that construction of the Shenyang intelligent elderly care platform was based on Internet of Things information technology and integrated resources such as families, communities, community health service centers, large medical service institutions, and government departments, but the platform does not involve online shopping, door-to-door meal delivery, online drug purchases, or other services.

### Future Perspectives

With the aging of society, there will be more apps suitable for the older population expounded. We should focus on the needs of older individuals when designing such apps. First, security issues are crucial. Users’ privacy and data security deserve our attention. We should also ensure that the services provided are safe. For example, if we design the function of online consultation, we should ensure the identity and qualification of the people who provide consultation services to older individuals. If we sell drugs for older individuals, we should ensure their authenticity and validity period. Second, we should consider the simplicity and convenience of the interface when designing the app. Most older individuals have poor vision, and if too many ads are inserted into the app or the interface is very complex, it will not be sufficiently user-friendly to facilitate use by the target population. We should use an interface with large font and simple functions to improve user comfort. In this way, we can ensure a high frequency of app use in the older population. In addition, when designing the app, we should consider the special needs of older individuals, such as one-click call, heart rate monitoring, and blood glucose records.

Finally, engineers should consider whether the design and promotion of the app should be combined with official elderly care institutions with the support of the government. Official agencies will increase the trust of users, but official agencies will make some requirements on the design of the app, which may limit some functions of the app.

### Study Implications

This study provides valuable knowledge for researchers, especially software engineers and health care professionals. With the aging population and the increasing use of smartphones among older individuals, smart elderly care apps will become the focus of attention. How to design an app that meets both the needs of older individuals and the requirements of medical staff is a problem that needs to be deeply explored by researchers. In China, very few studies have investigated the current status and needs of elderly care apps from the perspectives of both medical workers and older individuals Therefore, this study has practical significance, and an elderly care app designed based on the needs of users will be more consistent with the actual situation and market demand.

### Limitations

Although these results are consistent with previous data in the literature, some limitations should be considered when interpreting the results of this study. The study was not randomized, and the questionnaire responses and qualitative research were voluntary, which may result in limited sample representativeness. When analyzing the data, we found that no questionnaires were collected in Tibet, which is in line with our research findings that people in economically developed areas are more willing to be surveyed. However, this may lead to a certain bias in our research data.

Furthermore, this was a self-response survey, and although a pilot test was conducted with health care workers and older adults prior to formal recruitment, this design may lead to misinterpretation and missing data for some questions in the questionnaire.

In the qualitative interviews, most of the interviewees were interviewed by video calls because face-to-face interviews were very difficult during the COVID-19 epidemic. Although this does not violate the principles of qualitative research, compared with traditional face-to-face conversations, it may be more difficult to observe respondents’ microexpressions in video calls, which will also affect our interview results.

### Conclusion

In this study, medical staff and older adults surveyed in China reported a low usage rate of smart elderly care apps, but showed that the demand for such apps is huge. This demonstrates that it is necessary to develop and promote smart elderly care apps in China. Our survey found that when older individuals have chronic diseases, medical staff are more likely to recommend that they use smart elderly care apps to help them improve their health. Therefore, the design of the app should focus on the participation of medical staff so that they can better manage patients and promote the transformation of the doctor-patient relationship into a partnership. For participants who have not used such apps, the main reason for not using them is that they are not aware of their existence, followed by not knowing how to choose an app that suits them. This is a reminder that the publicity and promotion of apps are also very important. If the app can reach a wider population, it may be more effective. The results of the qualitative research suggest that the respondents most value a functional design, concise interface, and data security of apps. App developers should improve software design in these three areas to make their apps more accessible to the majority of people.

## Abbreviations

CNA: certified nursing assistant
OR: odds ratio

## Data Availability

The data sets generated during and/or analyzed during this study are not publicly available online but are available from the corresponding author on reasonable request.
